# Antidepressant-Like and Neuroprotective Effects of Ethanol Extract from the Root Bark of* Hibiscus syriacus* L.

**DOI:** 10.1155/2018/7383869

**Published:** 2018-11-19

**Authors:** Young Hwa Kim, A-Rang Im, Bo-Kyung Park, Seung Ho Paek, Goya Choi, Yu Ri Kim, Wan Kyunn Whang, Kang Hee Lee, Seung-Eun Oh, Mi Young Lee

**Affiliations:** ^1^Clinical Medicine Division, Korea Institute of Oriental Medicine, 1672 Yuseong-daero, Yuseong-gu, Daejeon 34054, Republic of Korea; ^2^Herbal Medicine Research Division, Korea Institute of Oriental Medicine, 1672 Yuseong-daero, Yuseong-gu, Daejeon 34054, Republic of Korea; ^3^Department of Life Science, Sogang University, 35 Baekbeom-ro, Mapo-gu, Seoul 04107, Republic of Korea; ^4^Pharmaceutical Botany Laboratory, College of Pharmacy, Chung-Ang University, 47 Heukseok-ro, Dongjak-gu, Seoul 06974, Republic of Korea; ^5^Department of Biological Sciences, Konkuk University, 120 Neungdong-ro, Gwangjin-gu, Seoul 05029, Republic of Korea

## Abstract

*Hibiscus syriacus *L. (Malvaceae) is an important ornamental shrub in horticulture and has been widely used as a medical material in Asia. The aim of this study was to assess the antidepressant and neuroprotective effects of a root bark extract of* H. syriacus *(HSR) and to investigate the underlying molecular mechanisms. Using an animal model of restraint stress, we investigated the effects of HSR on depressive-like behaviors and on the expression levels of serotonin, corticosterone, and neurotrophic factors in the brain. The mice were exposed to restraint stress for 2 h per day over a period of 3 weeks and orally treated with HSR (100, 200, or 400 mg/kg/day). We also examined the neuroprotective effect of HSR using corticosterone-treated human neuroblastoma SK-N-SH cells. The cells were incubated with the extract for 24 h, followed by corticosterone stimulation for 1 h, and then cell viability assay, cellular ATP assay, mitochondrial membrane potential (MMP) assay, cellular reactive oxygen species (ROS) assay, and western blotting were used to investigate the neuroprotective effects of HSR. Administration of HSR not only reduced the immobility times of the restraint-stressed mice in the forced swimming and tail suspension tests, but also significantly increased sucrose preference in the sucrose preference test. In addition, HSR significantly reduced the plasma levels of corticosterone and increased the brain levels of serotonin. The extract also increased the phosphorylation level of cyclic AMP response element-binding (CREB) protein and the expression level of brain-derived neurotrophic factor (BDNF). The in vitro assays showed that HSR pretreatment increased cell viability and ATP levels, recovered MMP, decreased ROS levels, and increased the expression of CREB and BDNF in corticosterone-induced neurotoxicity. Taken together, our data suggest that HSR may have the potential to control neuronal cell damage and depressive behaviors caused by chronic stress.

## 1. Introduction

Depression is an emotional disorder characterized by feelings of sadness, sleep disturbances, eating disorders, anxiety, and unexplained physical problems [1]. According to the 2017 World Health Organization report, more than 300 million people of all ages have depression worldwide [2].

It is difficult to understand depression pathology because various symptoms of depression cannot be explained under a single hypothesis. Currently recognized mechanisms, which aim at explaining the pathophysiology of depression, include monoamine hypothesis, genetic, environmental, immunological, and endocrine factors as well as neurosurgery [3]. Most people are exposed to a variety of stresses throughout their lives. If stress persists, it can adversely affect the body's immune system, cardiovascular system, central nervous system, and nerve transmission [4]. Chronic stress is a risk factor for developing symptoms in people with mild depression [5].

Currently available treatments for depression include tricyclic antidepressants, monoamine oxidase inhibitors, selective serotonin reuptake inhibitors (SSRIs), serotonin–norepinephrine reuptake inhibitors, various atypical antidepressants, and electroconvulsive therapy [6]. However, current antidepressant therapies are unsatisfactory because of their side effects [7, 8]. For this reason, many studies have been performed to find safe antidepressant drugs from natural sources.

*Hibiscus syriacus *L. (Malvaceae) is widely cultivated throughout eastern and southern Asia [9]. The flower, fruit, root, stem, and bark of* H. syriacus* all show pharmacological effects and have been widely used as medicinal treatment materials in Asia [10]. The root bark of* H. syriacus *has been used as a traditional medicine with fungicide, antipyretic, and anthelmintic activities in the treatment of dysentery, eczema, tinea, and scabies in Asia [11]. Recent studies have shown that the root bark of* H. syriacus* shows anticancer [12], antioxidant [9], and human neutrophil elastase inhibitory [13] activities. The antidepressant effects of some* Hibiscus* species have been investigated. The ethanol crude extract from the roots of* H*.* rosa-sinensis*, the ethanol extract of* H. sabdariffa* calyces, and the methanol extract from the flowers of* H. tiliaceus* showed significant antidepressant activity in animal models [14–16]. A coumarin analog and scopoletin, isolated from the methanol extract of* H*.* syriacus *root bark, showed monoamine oxidase inhibitory activity in mice [17]. For this reason, we aimed to investigate whether the ethanol extract of* H. syriacus* root bark had antidepressant effects in vitro and in vivo.

Therefore, we evaluated the effects of an ethanol extract from the root bark of* H. syriacus *(HSR) on depressive behaviors and the expression of neurotrophic factors in a mouse model of restraint stress-induced depression. Furthermore, we investigated whether the extract had neuroprotective effects on corticosterone-treated SK-N-SH human neuroblastoma cells and explored the underlying molecular mechanisms.

## 2. Materials and Methods

### 2.1. Preparation of H. syriacus Extract

The root bark of* H. syriacus *was collected at Yeongcheon-si (Gyungsangbuk-do, Korea) in September 2015 and was confirmed taxonomically by Dr. Go Ya Choi of the Korea Institute of Oriental Medicine (KIOM). A voucher specimen (C15070-5) has been deposited at the herbarium of the Clinical Medicine Division, KIOM (Daejeon, Korea). Dried and powdered root bark of* H. syriacus *(1.8 kg) was extracted with 10 volumes of 70% ethanol at 84°C for 3 h using a reflex extraction. The extract was concentrated and freeze-dried after filtration. The yield of the freeze-dried extract was approximately 7.18%.

### 2.2. High-Performance Liquid Chromatography Analysis of HSR

High-performance liquid chromatography (HPLC) analysis of HSR was performed on a system (Waters Corp., Milford, MA, USA) equipped with a 996-photodiode array detector (Waters Corp.). Ultraviolet absorbance was monitored at 200–400 nm. Two major compounds were quantified by integration of the peak areas at 230 nm. A column (Sunfire™-C_18_, 250 × 4.6 mm, 5 *μ*m; Waters Corp.) was maintained at 30°C. The mobile phase was composed of 0.2% phosphoric acid in water (solvent A) and acetonitrile (solvent B). The flow rate was 1.0 mL/min. The gradient conditions were as follows: 0–5 min, 30% to 40% B; 5–15 min, 40% to 60% B; and 15–30 min, 60% to 90% B. The injection volume was 10 *μ*L.

### 2.3. Animal Experiments

Seven-week-old male C57/BL6 mice were purchased from Samtako Bio, Inc. (Osan, Korea). Animal experiments were performed in accordance with the guidelines of the Animal Care and Use Committee at KIOM (KIOM-15-102). The mice were acclimated for 1 week and then kept under chronic restraint stress for 22 days (2 h/day) to induce depression. To effectively induce restraint stress, each mouse was placed in a well-ventilated plastic tube. After restraint stress induction, the mice were immediately returned to their home cage. The mice were randomly divided into the following groups (n = 6 per group): non-stress + saline (normal); stress + saline (control); stress + HSR 100 mg/kg (HSR 100); stress + HSR 200 mg/kg (HSR 200); stress + HSR 400 mg/kg (HSR 400); and stress + fluoxetine 20 mg/kg (FXT, positive control, Sigma–Aldrich, St. Louis, MO, USA) groups. The mice were orally administered either saline, HSR, or fluoxetine for 22 days and at 2 h before initiation of restraint stress. HSR and FXT were dissolved in saline solution before use.

### 2.4. Body Weight Changes and Sucrose Preference Test

The body weight was measured on days 1, 7, 14, and 21. A sucrose preference test (SPT) was used as a behavioral measure of anhedonia, a symptom of depression in rodents. The mice were housed two per cage and trained to consume a 2% (w/v) sucrose solution for 24 h before the test, with two bottles of a 2% sucrose solution placed in the cage. After 24 h of training, one sucrose solution bottle was replaced with a water bottle, and 24 h later, the positions of the bottles were switched to prevent the side-preference effect on the drinking behavior. Sucrose consumption was calculated as follows:

(1) Preference  %=sucrose  solution  intake  mLwater  intake  mL+sucrose  solution  intake  mL×100

The test was performed twice, on days 1 and 22, for 48 h each time. During the test, the mice had free access to both water and the sucrose solution.

### 2.5. Behavioral Tests

A forced swimming test (FST) was performed using the method described by Porsolt et al. [18]. The mice were allowed to preswim for 15 min on the day before the test and then were individually forced to swim in a cylinder (45 cm in height, 20 cm in diameter) filled with water (25 ± 2°C, 25 cm in depth). The total immobility time was measured during the last 4 min of the total swimming time (6 min) using video tracking software (EthoVision XT 9.0; Noldus Information Technology, Wageningen, Netherlands). A tail suspension test (TST) was performed by the method described by Steru et al. [19]. For TST, the mice, which were both acoustically and visually isolated, were suspended 50 cm above the floor using an adhesive tape placed approximately 1 cm from the tip of their tail. The immobility time was recorded during the last 4 min of a 6-min test using the same video tracking software as in FST.

### 2.6. Enzyme-Linked Immunosorbent Assay

Following anesthesia using Zoletil (25 mg/kg, Zoletil 50; Virbac, Cedex, France), the mice blood and brains were collected. Plasma was separated by centrifugation at at 4°C for 10 min (1,000 ×g). Plasma was stored at -80°C before use. Whole brain of each mouse was weighed and immediately homogenized on ice with 3-mL phosphate-buffered saline (PBS). The resulting homogenate was centrifuged at 4°C for 5 min (5,000 ×g). The supernatant was then used to determine serotonin levels. The plasma levels of corticosterone (Cayman Chemical Company, Ann Arbor, MI, USA) and the brain levels of serotonin were determined using enzyme-linked immunosorbent assay (ELISA) kits (Abcam, Cambridge, UK).

### 2.7. Cell Culture and Cell Viability

Human neuroblastoma SK-N-SH cells were obtained from the Korean Cell Line Bank (Seoul, Korea) and cultured in Dulbecco's modified Eagle's medium with 10% fetal bovine serum in an atmosphere of 5% CO_2_ at 37°C. Cells were plated at a density of 3 × 10^4^ cells/well in a 96-well plate and incubated at 37°C for 24 h. At 50% confluence, the cells in the treatment group were incubated with 0.25 mM corticosterone (Sigma–Aldrich) for 1 h. To evaluate the protective effects of HSR on corticosterone-treated SK-N-SH cells, HSR was dissolved in 5% dimethyl sulfoxide in Dulbecco's phosphate-buffered saline, and cells were pretreated with 10, 50, or 100 *μ*g/mL HSR for 24 h before the addition of corticosterone. Subsequently, cell viability was determined using a CellTiter Aqueous One Solution cell proliferation assay (Promega Corporation, Madison, WI, USA). Absorbance changes were detected at 490 nm using a microplate reader (Molecular Devices, San Jose, CA, USA).

### 2.8. Cellular ATP Measurement

Cells were treated with various concentrations of HSR for 1 h before the addition of 0.25 mM corticosterone. The total cellular ATP content was determined using an ATPlite luminescence assay kit (PerkinElmer, Waltham, MA, USA) and a TriStar LB 941 multimode microplate reader (Berthold Technologies, Calmbacher, Germany). The ATP content was determined using an internal standard and expressed as a percentage of the ATP content in untreated cells (control).

### 2.9. Caspase-3/7 Activity

Cells (5 × 10^4^ cells/well) were seeded in 96-well white plates and either left untreated or were pretreated with HSR for 1 h before the addition of 0.25 mM corticosterone. After 24 h, caspase-3 activity was measured using an Apo-ONE™ homogeneous caspase-3/7 assay kit (Promega Corporation) according to the manufacturer's specifications. The cells were lysed in 100 *μ*L of homogeneous caspase-3/7 buffer containing a caspase substrate, and the lysates were incubated for 1 h at room temperature. Caspase-3/7 activity was determined using a SoftMax Pro 5 fluorescence plate reader (Molecular Devices).

### 2.10. Mitochondrial Membrane Potential and Reactive Oxygen Species Measurements

A JC-1 kit (Biotium, Hayward, CA, USA) was used to detect changes in the mitochondrial membrane potential (MMP) as a surrogate indicator of mitochondrial function. Production of reactive oxygen species (ROS) was detected in corticosterone-treated SK-N-SH cells using carboxy-2′,7′-dichlorodihydrofluorescein diacetate (H_2_DCFDA), a cell-permeable fluorescent probe, according to the manufacturer's specifications (Sigma–Aldrich). Cells (3 × 10^4^ cells/well; 1 × 10^4^ cells/slide) were seeded in a 96-well white plate or in a chamber slide and treated with HSR for 24 h, followed by treatment with 0.25 mM corticosterone for 1 h. The media were aspirated from the plates, and the cells were washed with PBS. A volume of 100 *μ*L of the JC-1 reagent or H_2_DCFDA was added to each well, and the plates were incubated at 37°C for 20 min. The cells were washed twice with PBS, and then sufficient PBS was added to cover the cell layer. For the JC-1 detection, red fluorescence (excitation: 550 nm, emission: 600 nm) and green fluorescence (excitation: 485 nm, emission: 535 nm) were determined using a SoftMax Pro 5 fluorescence plate reader (Molecular Devices). The ratio of the red to green fluorescence is lower in dead cells and in cells undergoing apoptosis than in healthy cells. For the detection of H_2_DCFDA, green fluorescence (excitation: 485 nm, emission: 535 nm) was measured using the SoftMax Pro 5 fluorescence plate reader. The mitochondrial superoxide content was determined by incubation of cells with 5 *μ*M MitoSOX Red for 20 min at 37°C. MitoSOX Red fluorescence was measured using a confocal microscope (FV10i-LIV; Olympus, Tokyo, Japan). The excitation/emission wavelengths used for MitoSOX Red were 510/580 nm.

### 2.11. Quantitative Real-Time Polymerase Chain Reaction

Total RNA was extracted from 5 × 10^4^ cells using the TRIzol reagent (Invitrogen, Carlsbad, CA, USA) and quantified using a NanoDrop spectrophotometer (Thermo Fisher Scientific, Inc., Waltham, MA, USA). Quantitative real-time polymerase chain reaction (qRT-PCR) was performed using TaqMan assays (Applied Biosystems, Foster City, CA, USA) specific for interleukin (IL)-1*β*, IL-6, IL-8, and tumor necrosis factor (TNF)-*α* on a QuantStudio™ 6 Flex RT-PCR system (Applied Biosystems). Each sample was assayed in triplicate. Relative messenger RNA (mRNA) expression levels were calculated using the ΔΔCt method and normalized to those of *β*-actin in each sample.

### 2.12. Western Blot Analysis

Mouse brain samples were homogenized in 1 mL of lysis buffer (Pro-Prep™; iNtRON Biotechnology, Korea) containing 1 mM phenylmethylsulfonyl fluoride and a 1 *μ*g/mL protease inhibitor mixture. SK-N-SH cells were incubated with varying concentrations of HSR for 24 h at 37°C and then treated with 0.25 mM corticosterone for 1 h. The cells were harvested and lysed in lysis buffer containing a protease inhibitor mixture. Equal amounts of proteins were separated by 10% sodium dodecyl sulfate polyacrylamide gel electrophoresis and transferred to polyvinylidene difluoride membranes (Amersham Biosciences, Piscataway, NJ, USA), which were blocked with 5% skim milk in Tris-buffered saline with 0.1% Tween 20 for 1 h. The membranes were probed overnight with specific primary antibodies against BDNF, phosphorylated cyclic AMP response element-binding (CREB) protein (p-CREB), CREB, extracellular signal-regulated kinase (ERK), p-ERK, c-Jun N-terminal kinase (JNK), p-JNK, p-p38, and p38 (Cell Signaling Technology, Inc., Danvers, MA, USA) at 4°C. Then, the blots were incubated with a horseradish peroxidase-conjugated secondary antibody for 2 h at room temperature. Immunoreactive bands were detected using a chemiluminescent detection reagent (Amersham Biosciences) and visualized using an ImageQuant LAS 4000 Mini imager (GE Healthcare). *β*-Actin (Santa Cruz Biotechnology, Inc., Dallas, TX, USA) was used as a loading control.

### 2.13. Statistical Analysis

All experimental data are presented as the mean ± standard deviation (SD). Multiple group comparisons were performed using one-way analysis of variance, followed by post hoc Tukey's test. The GraphPad Prism 7 software (GraphPad Software, Inc., La Jolla, CA, USA) was used for the analysis, and a* p*-value of < 0.05 was considered statistically significant.

## 3. Results

### 3.1. HPLC Analysis of HSR

**Figure 1:**
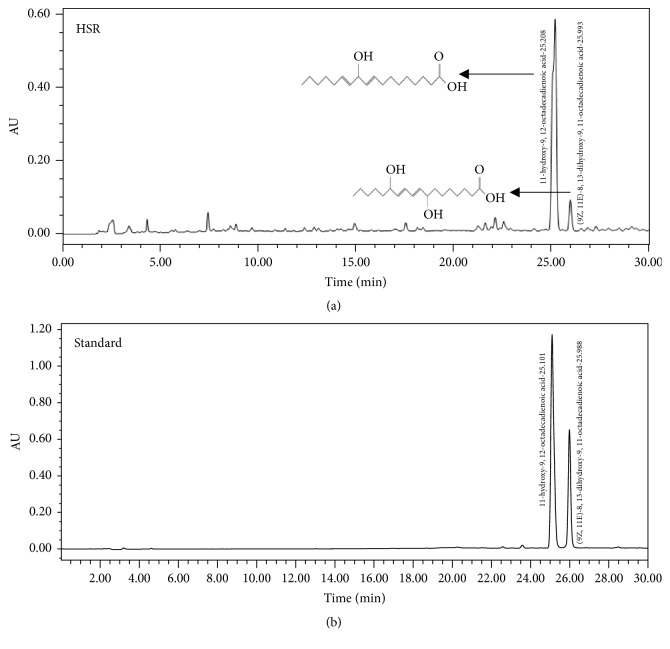
HPLC analysis of HSR. (a) HPLC chromatogram of HSR. (b) HPLC chromatogram of 11-hydroxy-9,12-octadecadienoic acid and (9Z,11E)-8,13-dihydroxy-9,11-octadecadienoic acid.

Chromatography was performed to separate a single component from HSR. 11-Hydroxy-9,12-octadecadienoic acid and (9Z,11E)-8,13-dihydroxy-9,11-octadecadienoic acid were separated from HSR. These two components were verified by HPLC analysis. This analysis provided chemical information about HSR and was performed to ensure reproducibility of our experiment when using various batches of HSR. The HPLC chromatogram of HSR showed peaks of 11-hydroxy-9,12-octadecadienoic acid and (9Z,11E)-8,13-dihydroxy-9,11-octadecadienoic acid with retention times of 25.208 and 25.993 min, respectively (Figure 1).

### 3.2. Effects of HSR on Body Weight Changes and Sucrose Preference

**Figure 2:**
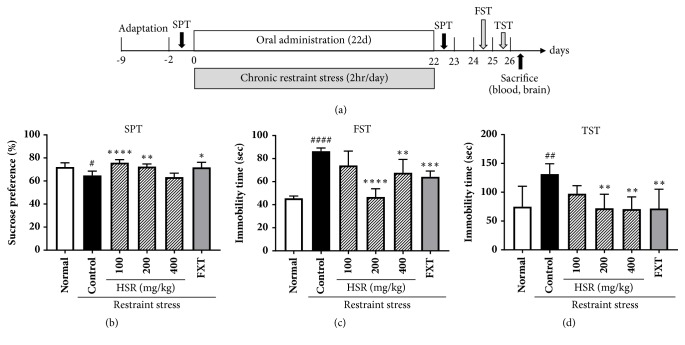
Effects of HSR on depression-related behaviors in restraint stress-induced mice. (a) Animal experimental protocol. The mice were orally administered vehicle (saline), HSR (100, 200, or 400 mg/kg), or fluoxetine (FXT; 20 mg/kg) daily for the indicated period. Restraint stress and oral administration schedules for behavioral experiments are presented. FST: forced swimming test; SPT: sucrose preference test; TST: tail suspension test. (b) Sucrose preference was measured on the indicated days. The immobility time in (c) FST and (d) TST was measured on day 23. Data are the mean ± SD (*n* = 6). ^#^*p* < 0.05, ^##^*p* < 0.01, and ^####^*p* < 0.0001 vs. the normal group; ^*∗*^*p* < 0.05, ^*∗∗*^*p* < 0.01, ^*∗∗∗*^*p* < 0.001, and ^*∗∗∗∗*^*p* < 0.0001 vs. the control group.

Our experimental design, including restraint stress and administration schedule, is presented in Figure 2(a). The mean body weights were initially comparable across the six groups of mice. After restraint stress, the control group demonstrated decreased body weight on days 14 and 21 compared with that in the normal group; however, no differences were observed in the body weight changes between the control and HSR groups (data not shown). Before the restraint stress, the mice in all groups showed no differences in their sucrose preference (data not shown). However, after 22 days of applying restraint stress, sucrose preference in the control group was significantly suppressed compared with that in the normal group. HSR (100 and 200 mg/kg) and FXT treatments increased sucrose preference compared with that in the control group (*p* < 0.0001,* p* < 0.01, and* p* < 0.05, resp.; Figure 2(b)). These results suggest that HSR may recover the anhedonia loss without affecting the body weight change in restraint stress-induced mice.

### 3.3. Effects of HSR on Depression-Like Behaviors

On the day after SPT was completed, FST and TST were performed. As expected, the immobility time in FST was significantly longer in the control group (86.33 ± 2.875 seconds) than in the normal group (45.5 ± 2.074 seconds;* p* < 0.0001; Figure 2(c)). In the HSR-treated groups, the immobility times markedly diminished compared with that in the control group (HSR 200:* p* < 0.0001; HSR 400:* p* < 0.01; Figure 2(c)). In TST, the immobility times were shorter in the HSR-treated groups (HSR 200: 72.0 ± 24.57 seconds; HSR 400: 70.67 ± 21.29 seconds; both* p* < 0.01) than that in the control group (131.3 ± 18.01 seconds; Figure 2(d)). These results suggest that HSR may control depression-like behaviors in restraint stress-induced mice.

### 3.4. Effects of HSR on Stress-Related Hormones in Restraint Stress-Induced Mice

**Figure 3:**
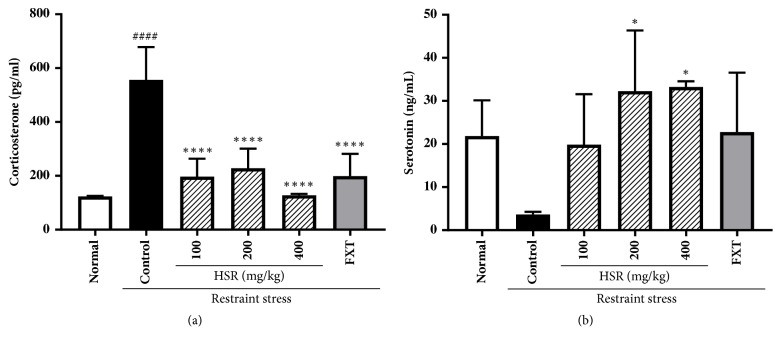
Effects of HSR on plasma levels of corticosterone (a) and brain levels of serotonin (b) in restraint stress-induced mice. Data are the means ± SD (*n* = 4, corticosterone; n = 3, serotonin). ^####^*p* < 0.0001 vs. the normal group; ^*∗*^*p* < 0.05 and ^*∗∗∗∗*^*p* < 0.0001 vs. the control group.

The levels of the stress hormone corticosterone significantly increased in the control mice; however, the HSR- and FXT-treated mice showed greatly reduced levels compared with those in the control mice (Figure 3(a)). The brain levels of serotonin, an indicator of mood disorder, significantly decreased in restraint stress-induced mice; however, oral administration of HSR dose-dependently reversed this suppression, leading to above-normal serotonin levels (Figure 3(b)). FXT also increased serotonin production. These data suggest that HSR may control stress-related hormones in restraint stress-induced mice.

### 3.5. Effects of HSR on BDNF and p-CREB Expression in the Brain

**Figure 4:**
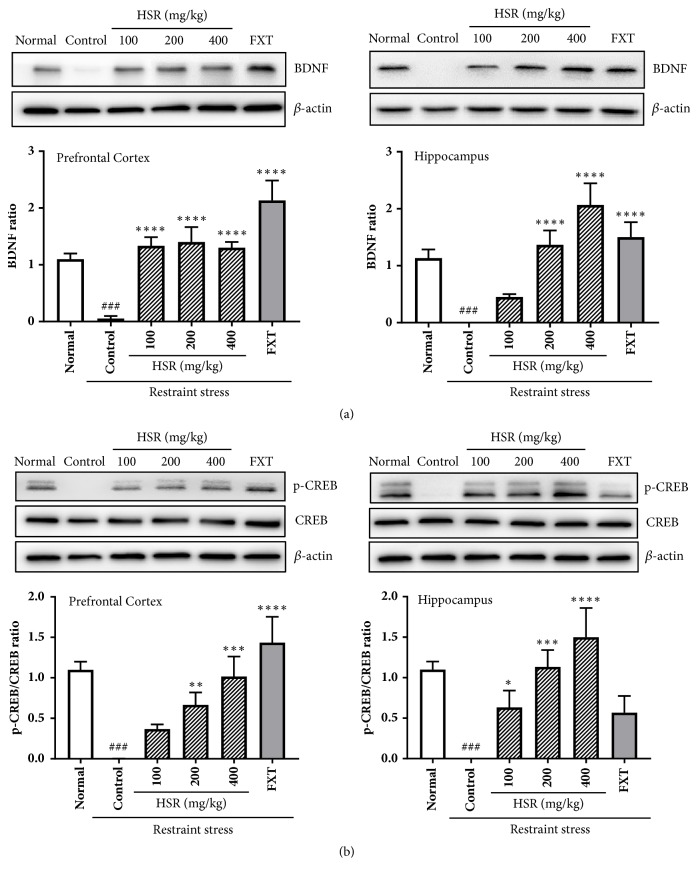
Effects of HSR on BDNF and p-CREB/CREB expression in the brain of restraint stress-induced mice. Lysates of isolated prefrontal cortex and hippocampus were analyzed by western blotting using (a) BDNF and (b) p-CREB/CREB antibodies. *β*-Actin was used as the loading control. The data are representative of three independent experiments. Data are the mean ± SD (*n* = 3). ^###^*p* < 0.001 vs. the normal group; ^*∗*^*p* < 0.05, ^*∗∗*^*p* < 0.01, ^*∗∗∗*^*p* < 0.001, and ^*∗∗∗∗*^*p* < 0.0001 vs. the control group.

To understand the effects of HSR on depression-like symptoms at the molecular level, BDNF and p-CREB expression were examined in brain regions by western blot analysis. The BDNF levels were dramatically lower in the control group than those in the normal group, indicating neurotoxic damage to the brain; however, HSR treatment significantly enhanced the BDNF levels in a dose-dependent manner in the prefrontal cortex and hippocampus (Figure 4(a)). Downregulation of CREB activity is associated with major depressive disorders. The level of p-CREB also decreased in the brain of the control group and markedly increased in the HSR- and FXT-treated groups (Figure 4(b)). These results suggest that HSR may affect the neuronal activity in the brains of restraint stress-induced mice.

### 3.6. Anticytotoxicity of HSR for Corticosterone-Treated SK-N-SH Cells

**Figure 5:**
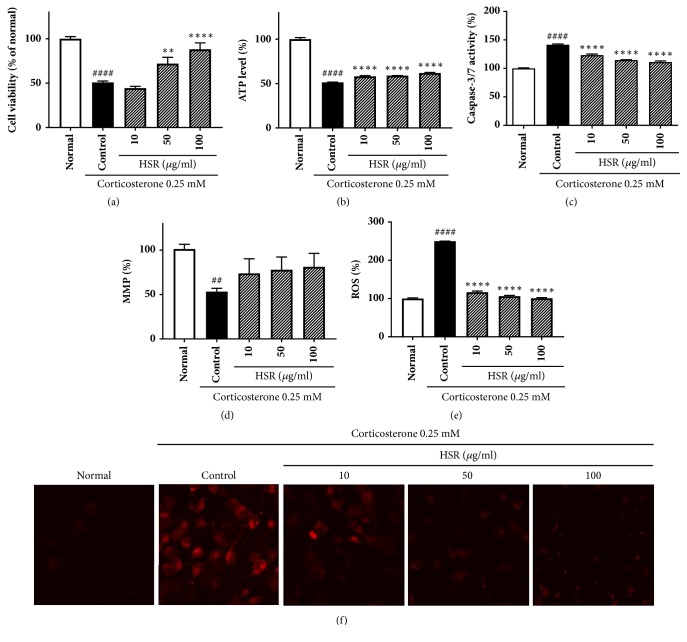
Effects of HSR on cell viability, mitochondrial function, and mitochondrial superoxide generation in SK-N-SH cells. Cells were pretreated with 0, 10, 50, and 100 *μ*g/mL HSF for 1 h before the addition of 0.25 mM corticosterone. (a) Cell viability. (b) ATP levels. (c) Apoptotic caspase-3/7 activity. (d) The mitochondrial membrane potential (MMP) was measured using JC-1 fluorescence. (e) Reactive oxygen species (ROS) generation was measured by H_2_DCFDA staining. (f) Mitochondrial superoxide production was measured by confocal microscopy (60 × 1.0). Data are expressed as a percentage of control. Data are the mean ± SD (*n* = 3 per group). ^##^*p* < 0.01 and ^####^*p* < 0.0001 vs. the normal group; ^*∗∗*^*p* < 0.01 and ^*∗∗∗∗*^*p* < 0.0001 vs. the control group.

The protective effects of HSR on SK-N-SH cells were quantitatively evaluated using the 3-(4,5-dimethylthiazol-2-yl)-5-(3-carboxymethoxyphenyl)-2-(4-sulfophenyl)-2*H*-tetrazolium cell viability assay after treating cells with various concentrations of corticosterone (0.05, 0.1, 0.25, 0.5, 0.75, and 1 mM). The data showed that 0.25 mM corticosterone reduced the cell viability to up to 50% of that of the untreated cells (Figure 5(a)). Pretreatment with HSR at different concentrations (10, 50, and 100 *μ*g/mL) for 1 h before the exposure to corticosterone increased cell viability to 60–80% of that of the normal cells. The results show that HSR may inhibit the corticosterone-induced neurotoxicity.

### 3.7. Effects of HSR on Total Cellular ATP Levels and Caspase-3/7 Activity

ATP is an indicator of cell viability as it exists in every active cell of matter. Because ATP concentration declines rapidly when cells undergo necrosis or apoptosis, monitoring ATP level is a good indicator of cytocidal, cytostatic, and proliferation effects. To determine whether HSR affects the energy production, we measured total cellular ATP concentrations after cell treatment with the extract. As shown in Figure 5(b), the ATP production was lower in the corticosterone-treated cells than that in the control cells. HSR pretreatment increased the ATP production compared with that in the cells treated with corticosterone alone (Figure 5(b)). Moreover, 1-h treatment with 0.25 mM corticosterone resulted in a 141.6% increase in caspase-3/7 activity compared with that in the control cells. Cells pretreated with HSR exhibited a reduced caspase-3/7 activity compared with that in the cells treated with corticosterone alone (Figure 5(c)).

### 3.8. Effects of HSR on MMP and ROS

We used a red/green fluorescence intensity ratio (JC-1 ratio) to assess the effects of HSR on corticosterone-induced changes in MMP. A decrease in the JC-1 ratio indicates the MMP depolarization. Cells treated with 0.25 mM corticosterone had a significantly lower JC-1 ratio than that in the normal cells. Moreover, cells pretreated with HSR had a significantly higher JC-1 ratio than that in the cells treated with corticosterone alone (Figure 5(d)). H_2_DCFDA staining was performed to measure the ROS levels, and the data revealed that the ROS generation increased in corticosterone-treated cells but was attenuated in cells pretreated with HSR (Figure 5(e)). Next, the MitoSOX Red mitochondrial superoxide indicator was used to evaluate the superoxide production. Corticosterone increased the mitochondrial superoxide level. However, pretreatment with HSR decreased superoxide level (Figure 5(f)).

### 3.9. Effects of HSR on the Expression of Proinflammatory Cytokines

**Figure 6:**
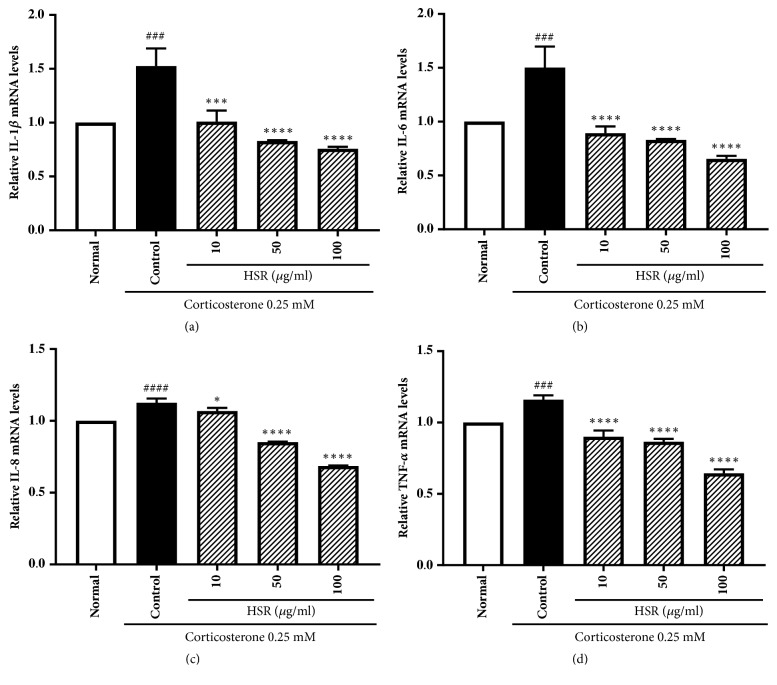
Effects of HSR on corticosterone-induced proinflammatory cytokine expression. Total RNA was extracted from SK-N-SH cells, and the levels of (a) IL-1*β*, (b) IL-6, (c) IL-8, and (d) TNF-*α* mRNA were determined by qRT-PCR. Expression levels of the target genes were normalized to those of *β*-actin. Data are the mean ± SD (*n* = 3). ^###^*p* < 0.001 and ^####^*p* < 0.0001 vs. the normal group; ^*∗*^*p* < 0.05, ^*∗∗∗*^*p* < 0.001, and ^*∗∗∗∗*^*p* < 0.0001 vs. the control group.

Proinflammatory cytokines such as IL-1*β*, IL-6, IL-8, and TNF-*α* play important roles in neuroinflammation and neuronal death. Corticosterone-treated SK-N-SH cells showed higher mRNA expression levels of IL-1*β*, IL-6, IL-8, and TNF-*α* than those in the normal cells. In contrast, cells pretreated with HSR exhibited reduced mRNA expression levels compared with those in the cells treated with corticosterone alone (Figures 6(a)–6(d)).

### 3.10. Promotion of BDNF and CREB Expression by HSR

**Figure 7:**
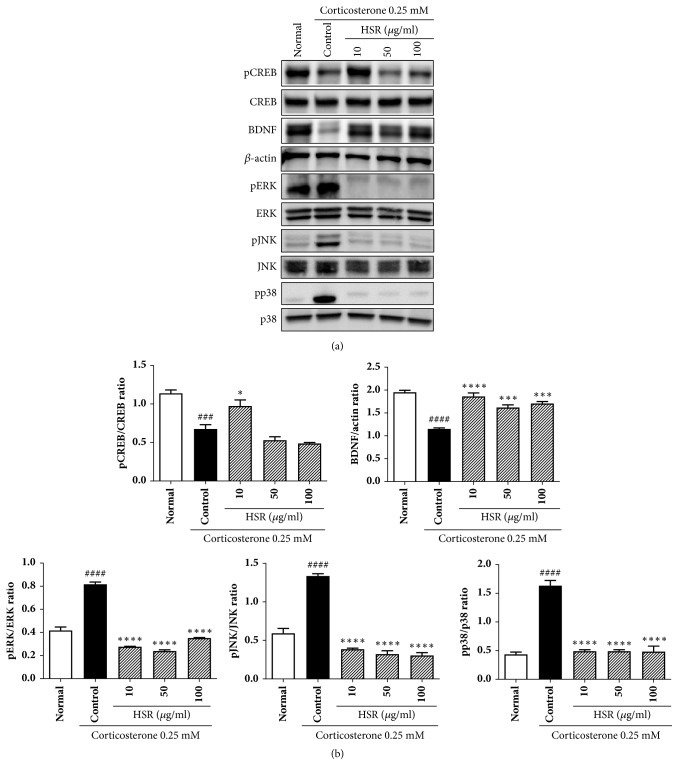
Effects of HSR on protein expression of BDNF, CREB, and MAPKs in corticosterone-treated SK-N-SH cells. (a) Western blotting analysis and (b) intensity of protein bands were quantified by densitometry. Total CREB, ERK, JNK, p38, and *β*-actin were used as internal standards. The data are representative of three independent experiments. Data are the mean ± SD (*n* = 3). ^###^*p* < 0.001 and ^####^*p* < 0.0001 vs. the normal group; ^*∗*^*p* < 0.05, ^*∗∗∗*^*p* < 0.001, and ^*∗∗∗∗*^*p* < 0.0001 vs. the control group.

Corticosterone-treated cells showed reduced BDNF and CREB expression and increased mitogen-activated protein kinase (MAPK) expression compared with that in normal cells. In contrast, cells pretreated with HSR showed increased BDNF and CREB expression and reduced MAPK expression compared with that in the cells treated with corticosterone alone (Figures 7(a) and 7(b)).

## 4. Discussion

Chronic stress models are most commonly used in animal studies to mimic the biology and pathology of human depressive disorders [20, 21]. Restraint stress, which is induced by restraining laboratory animals for a certain period of time, is the most commonly used method for generating a chronic stress model [22–25]. In this study, we evaluated the effects of HSR using an animal model of chronic stress. Anhedonia, or the decreased ability to experience pleasure, is one of the major symptoms of depression, which can be tested using SPT [26]. Our results showed that sucrose preference decreased when the mice were under restraint stress but increased when the mice were administered HSR. Thus, HSR could restore the ability of depressed mice to experience pleasure. Similar results were observed in FST and TST, which are the most widely used tests to evaluate antidepressant action and to infer a depression-like behavior [27]. Administration of HSR reduced the immobility time in FST and TST. However, not all behavioral tests were dependent on the concentration of HSR extracts. A concentration of 100 mg/kg worked best in SPT and 200 mg/kg in FST. The effects of 200 and 400 mg/kg were similar in TST. Taken together, the optimum concentration of HSR to improve depression was estimated at 200 mg/kg in behavior test. These results may have been associated with the pharmacokinetic properties of HSR, which comprises various compounds. Pharmacokinetic properties are often unpredictable in multi compound herbal medicines [28]. Nevertheless, HSR is believed to have the potential to be developed as an antidepressant.

The HPA axis is considered an important target for the prevention and treatment of depression. Chronic exposure to restraints may disrupt the HPA axis and elevate the levels of plasma corticosterone [28]. In the present study, HSR administration significantly reduced the restraint stress-induced increase in corticosterone levels, similar to FXT treatment. These results support the hypothesis that the mechanism underlying the antidepressant action of HSR is associated with the HPA axis.

Serotonin (5-hydroxytryptamine, 5-HT) is a potent neurotransmitter whose levels in the central nervous system are closely linked to emotional disorders, including depression [29–31]. In this study, brain serotonin levels decreased following 3 weeks of restraint stress, but these levels significantly and rapidly increased in the HSR-treated groups.

BDNFs are essential for the survival, growth, and maintenance of neurons in core brain circuits associated with emotional and cognitive functions [32]. CREB, a nuclear transcription factor, is a key player in multiple intracellular signaling pathways. During neuronal stimulation, CREB is normally activated by phosphorylation at Ser-133, which is involved in the pathology of depression [33–35]. Previous studies have demonstrated that long-term treatment with classical antidepressants such as SSRIs increases BDNF mRNA transcription by promoting CREB protein phosphorylation [8, 35, 36]. Our data showed that BDNF levels significantly decreased in the cortex and hippocampus of the control group of mice and returned to normal levels in the HSR groups. The p-CREB/CREB ratio also dramatically increased upon HSR administration. These results confirmed that HSR exerted antidepressant-like effects via the CREB/BDNF signaling pathway.

In addition, we examined whether the antidepressant effects of HSR involve the inhibition of oxidative stress and cell apoptosis. Corticosterone, a well-known stress hormone, is the main stimulus used to establish in vitro and in vivo models of major depression disorders [37]. In this study, we tested the neuroprotective effects of HSR against corticosterone-induced neurotoxicity using SK-N-SH cells. Pretreatment with HSR, prior to treatment with 0.25 mM corticosterone, increased cell viability in a dose-dependent manner compared with that in corticosterone-only treatment group. These results suggested that HSR could increase cell viability and protect cells from corticosterone-induced neurotoxicity.

Glucocorticoids such as corticosterone play an important biphasic role in modulating neural plasticity and affecting mitochondrial function. As a result, continuous exposure to glucocorticoids triggers apoptosis [38, 39]. Consistent with this evidence, we found that corticosterone treatment decreased the cellular ATP levels and increased caspase-3/7 activity. However, pretreatment with HSR attenuated these changes, indicating that HSR induces mitochondrial protection in vitro. It has been demonstrated that corticosterone induces apoptosis through activation of the caspase-3 apoptosis pathway [40]. Our findings indicate that HSR may exert its therapeutic effects by regulating the mitochondrial dysfunction.

Corticosterone has also been shown to impair mitochondrial function and promote oxidative stress in the brain [39]. Disturbance of the balance between the production of ROS and antioxidant defense systems may contribute to corticosterone-induced neuronal injury [41, 42]. We found that HSR reversed the corticosterone-induced generation of ROS and the loss of MMP in SK-N-SH cells. These results suggest that the antidepressant effects of HSR are associated with the inhibition of neurotoxicity caused by corticosterone.

External stressors may induce (neuro)inflammation, neurodegeneration and reduce neurogenesis [43]. In this study, HSR reduced the expression of proinflammatory cytokines such as IL-1*β*, IL-6, IL-8, and TNF-*α*. Thus, the anti-inflammatory effects of HSR can be attributed to its direct suppressive effects on the expression of proinflammatory cytokines.

Additionally, HSR was found to promote the expression of CREB, which enhances the transcription of many target genes, including BDNF, to influence structural plasticity and produce trophic effects in neurons [44]. We also found that pretreatment with HSR prior to corticosterone stimulation decreased the corticosterone-induced MAPK expression. MAPKs, including ERK-1/2, p38, and JNK, are important for normal adult brain functions, including synaptic plasticity, learning, and memory [45]. These results suggested that HSR protected SK-N-SH cells from corticosterone-induced cytotoxicity by inhibiting MAPK activation and promoting CREB activity and subsequently revealed the relationship between stress and depression. In summary, HSR can be used as a neuroprotective agent and may potentially be used as a therapy for stress-induced depression.

Traditional herbal medicines are often a mixture containing a variety of individual compounds. Various extraction procedures produce different compounds with different effects. An acetone extract from the* H. syriacus *root bark has been reported to exhibit anticancer activity [10–12]. Hydroxyhibiscone A, which showed moderate activity as a human neutrophil elastase inhibitor, was isolated from a methanol extract of* H. syriacus* [13]. However, there are no reports on the ethanol extracts of* H. syriacus *root bark. In our study, 11-hydroxy-9,12-octadecadienoic acid and (9*Z*,11*E*)-8,13-dihydroxy-9,11-octadecadienoic acid were isolated from the root bark of* H. syriacus *using ethanol extraction. Further study on these compounds is needed.

## 5. Conclusions

This study showed that administration of HSR significantly reduced the depression-like behavior by activating the CREB/BDNF signaling pathway in a mouse model of depression induced by restraint stress. In addition, HSR reduced the cytotoxicity of corticosterone via inhibition of the MAPK pathway and, therefore, reduced the mitochondrial oxidative stress and neuronal inflammation associated with this pathway. These results suggest that HSR may have the potential to be used as an antidepressant to control depressive behaviors and neuronal cell damage caused by chronic stress.

## Data Availability

The datasets used and/or analyzed in the current study are available from the corresponding author upon reasonable request. The role of the funding body in the design of the study and collection, analysis, and interpretation of data and in writing the manuscript should be declared in this request.

## Abbreviations

BDNF: Brain-derived neurotrophic factor
CREB: Cyclic AMP response element-binding protein
ELISA: Enzyme-linked immunosorbent assay
ERK: Extracellular signal-regulated kinase
FST: Forced swimming test
FXT: Fluoxetine
H_2_DCFDA: Carboxy-2′,7′-dichlorodihydrofluorescein diacetate
HPA: Hypothalamic–pituitary–adrenal
HPLC: High-performance liquid chromatography
HSR: Root bark extract of* Hibiscus syriacus*
5-HT: 5-Hydroxytryptamin
IL: Interleukin
JNK: c-Jun N-terminal kinase
KIOM: Korea Institute of Oriental Medicine
MAPK: Mitogen-activated protein kinase
MMP: Mitochondrial membrane potential
mRNA: Messenger RNA
p-: Phosphorylated
PBS: Phosphate-buffered saline
qRT-PCR: Quantitative real-time polymerase chain reaction
ROS: Reactive oxygen species
SD: Standard deviation
SPT: Sucrose preference test
SSRI: Selective serotonin reuptake inhibitor
TNF: Tumor necrosis factor
TST: Tail suspension test.
